# Same ion channel populations and different excitabilities: beyond the conductance-based model

**DOI:** 10.1186/1471-2202-13-S1-P120

**Published:** 2012-07-16

**Authors:** Marco Arieli Herrera Valdez

**Affiliations:** 1 Department of Mathematics, University of Arizona, Tucson, AZ, 85719, USA; 2 Department of Mathematics and Physics, University of Puerto Rico at Cayey, Cayey, PR, 00736, USA; 3 Institute of Interdisciplinary Research, University of Puerto Rico at Cayey, Cayey, PR, 00736, USA

## 

Multicellular organisms rely on electrical signaling to communicate messages within and between different tissues. Channel-mediated ionic transport is typically modeled with conductance-based formulations that assume currents are the result of ionic electrical drift, without taking diffusion into consideration [1]. In contrast, formulations of current that assume ionic flux depends on electrical drift and diffusion are not as widely used in the literature [3-5]. These representations are more realistic and display experimentally observable phenomena that conductance-based models cannot reproduce (e.g. rectification). The two formulations are, however, mathematically related because conductance-based currents are linear approximations of drift-diffusion currents. Importantly, conductance-based models of membrane potential are not first order approximations of drift-diffusion models.

The two approaches predict qualitatively and quantitatively different behaviors in the dynamics of membrane potential. For instance, two neuronal membrane models with identical populations of ion channels, one written with conductance-based currents, the other with drift-diffusion currents, undergo transitions into and out of repetitive oscillations through different mechanisms and for different levels of stimulation. These differences in excitability are observed across different levels of ion channel expression in response to excitatory synaptic input. The electrophysiological profiles of drift-diffusion and conductance-based models having identical ion channel populations are different. As a consequence, the input-output and computational properties of networks constructed with conductance-based and electrodiffusion models should be different as well. The drift-diffusion formulation is proposed as a theoretical improvement over conductance-based models that may lead to more accurate predictions and interpretations of experimental data at the single cell and network levels.
